# Stabilization of Fish Protein-Based Adhesive by Reduction of Its Hygroscopicity

**DOI:** 10.3390/polym16152195

**Published:** 2024-08-01

**Authors:** Branka Mušič, Jaka Gašper Pečnik, Andreja Pondelak

**Affiliations:** 1 Slovenian National Building and Civil Engineering Institute, Dimičeva Ulica 12, 1000 Ljubljana, Slovenia; branka.music@zag.si; 2 InnoRenew CoE, Livade 6, 6310 Izola, Slovenia; jaka.pecnik@innorenew.eu

**Keywords:** protein-based adhesive, polymer stabilization, hygroscopicity, fish adhesive modification, fish industry waste, circular economy, bonding properties

## Abstract

Protein-based fish adhesives have historically been used in various bonding applications; however, due to the protein’s high affinity for water absorption, these adhesives become destabilized in high-moisture environments, resulting in reduced bondline strength and early failure. This limitation makes them unsuitable for industrial applications with higher demands. To address this issue, water-insoluble raw powder materials such as iron, copper, or zeolite were incorporated into natural fish adhesives. In this study, the hygroscopicity, dry matter content, thermal analysis (TGA/DSC), FT-IR spectroscopy, surface tension measurements, vapour permeability, and scanning electron microscope (SEM) of the modified adhesives were determined. In addition, the bonding properties of the modified adhesives were evaluated by the tensile shear strength of the lap joints, and mould growth was visually inspected. The resulting modified protein-based adhesives demonstrated improved stability in high humidity environments. Enhancing the hygroscopic properties of protein-based fish adhesives has the potential to unlock new opportunities and applications, providing a healthier and more environmentally sustainable alternative to petroleum-based adhesives.

## 1. Introduction

Wood has a long tradition as a building material. Over the last two decades, advancements in technology have brought about vast improvements in the wooden construction sector, enabling the construction of taller, more affordable, and higher quality buildings. Additionally, wood has gained renewed popularity due to its environmental benefits, especially in the face of increasing pollution and climate change [1]. Given the size limitations of sawn timber, glued laminated timber is commonly used in timber construction, alongside other engineered timber products. Adhesives are a key component in producing these engineered wood products, as they must withstand high structural demands under extreme conditions and fluctuations in temperature and humidity. Currently, petroleum-based adhesives are predominantly used in timber products. For example, toxic formaldehyde-based adhesives, such as phenol-formaldehyde, urea-formaldehyde, melamine formaldehyde, and melamine-urea-formaldehyde, as well as other solvent-based adhesives like polyurethanes and epoxy adhesives [2], are commonly used in wood composites. These adhesives pose potential health risks due to the emission of VOCs, which has created a high demand for sustainable and non-toxic bio-based adhesives [3,4].

Protein-based structural biomaterials have been investigated for various applications because the sequence flexibility within the proteins may improve their mechanical and structural integrity [5]. This is an important area of research as wood-based construction is on the rise, and the market for wood adhesives and binders is predicted to reach over USD 21 billion by 2024 [6]. However, the use of protein-based adhesives is limited to a specific context, encompassing materials exclusively from a natural, non-mineral source which can be modified to obtain the properties required to substitute synthetic adhesives [7,8,9,10].

Adhesives such as soy and fish protein have become more attractive as they are healthier, economically viable within a circular economy, and more environmentally friendly and sustainable compared to petroleum-based adhesives [3,11]. The marine industry’s side streams and biomass waste offer a potentially abundant source of protein [12]. Numerous studies have already been carried out on these adhesives obtained from renewable sources. For example, soy proteins for gluing wood-based materials have been studied; however, these studies showed that the water resistance of these adhesives must be improved [13,14,15]. Other sources of biomass such as lignin [16,17,18], starch [19,20], tannins [21,22], vegetable oils [23], and proteins [24,25] have been studied as well. The latter are the most abundant class of macromolecules within bio-based materials as they constitute the main organic building blocks in living organisms.

Fish protein-based adhesives (fPBA) are non-toxic, biodegradable, and soluble in water, making them suitable for all applications that require a combination of high elasticity and very high strength. They exhibit strong adhesion to wood, ceramic, and metal. However, bio-based adhesives exhibit 20% lower shear strength compared to synthetic ones, and after wetting there is an additional one-third decrease compared to the original value [26]. In addition to the bonding ability, the stability of protein-based adhesives (PBA) in various environments is also crucial. The stability of these natural polymers is highly dependent on humidity levels, which makes them unsuitable for industrial use. Protein molecules tend to absorb water, which destabilizes the natural polymer, weakens the bond strength, and leads to early failure [27,28]. Therefore, an innovative approach to increase the adhesive’s resistance to high ambient humidity is required for stabilizing and promoting sustainable and healthy adhesives in the wood industry.

While protein-based adhesives contain hydroxyl groups and have limited scope, chemically modified adhesives can exhibit superior water resistance in adhesive applications. These modifications include various processes such as etherification, crosslinking, grafting, oxidation, or the utilisation of bio-based coupling agents to prepare modified and stabilized adhesives [29], etc. There have been some attempts to improve the hydrophobicity of fish gelatine; for example, in one study, the surface hydrophobicity index of fish-scale gelatine was increased by phosphorylation [30]. However, a systematic study on “blue growth” [12] modifications is lacking, particularly in relation to the modification pathway of fish adhesives and the assessment of their functional and physio-chemical properties [30,31]. Generally, adding powdered raw materials to polymer materials serves several purposes, such as improving their mechanical properties (e.g., tensile strength, stiffness, and impact resistance), reducing the overall cost of the polymer product, enhancing its performance, and acting as barrier particles, which increase their stability and resistance to various environmental factors while extending the product’s lifespan. The choice of barrier filler depends on the specific requirements of the application, specific environmental challenges the polymer product will face, and the desired properties of the final polymer product. These properties include providing the necessary water resistance while maintaining the desired mechanical and functional properties. Some powdered materials, such as metal powders (Fe, Cu), or additives like microporous powder (zeolite), can be used to create gas and moisture barriers in polymers by preventing the penetration of gases like oxygen or water vapour through the polymer film.

Copper is a well-known antimicrobial agent [32,33,34]. In 2008, the United States Environmental Protection Agency (US EPA) officially recognised copper as an antimicrobial metal [35]. Copper does not contain any components that are either persistent, bio-accumulative, and toxic (PBT) or very persistent and very bio-accumulative (vPvB) at levels of 0.1% or higher.

Iron reacts with water to form iron (III) oxide and hydrogen. Both iron powder and iron oxide exhibit antimicrobial properties [36,37,38]. Additionally, their magnetic properties enable their use in small crevices with a magnet, allow for remote manipulation via an external magnetic field, which facilitates position monitoring, and even enable the use of heat to accelerate drying/hardening.

Zeolite is an aluminosilicate mineral (Ca,K_2_,Na_2_,Mg)_4_ Al_8_Si_40_O_96_·24H_2_O with several industrial, agricultural, and medical uses. Clinoptilolite is one of the most abundant natural zeolites. It has a microporous skeletal structure composed of aluminium [AlO_4_] and silicon [SiO_4_] tetrahedrons connected by common oxygen atoms which provides open voids in the form of cages and tubules that can adsorb various substances [39]. Additionally, clinoptilolite is ecologically and environmentally friendly.

This paper presents three types of modifications where powder barrier fillers (Cu, Fe or zeolite) were added to fish protein-based adhesives. Furthermore, this study investigated the effect of elevated humidity on samples of modified fish protein-based adhesives compared to the original fPBA. Analytical methods such as TGA, DSC, SEM, FT-IR, and surface tension measurements were used; in addition, the sensitivity of the tested samples to mould growth and their ability to prevent water vapour permeability were investigated.

## 2. Materials and Methods

### 2.1. Materials

fPBA in granulate form was purchased from Kremer Pigmente GmbH & Co. KG (Aichstetten, Germany). Different powder raw materials containing elements were incorporated into the fPBA, including copper (Cu), iron (Fe), and zeolite (Z). Cu (99.7%; Merck KGaA, Darmstadt, Germany) had a particle size < 63 μm, and Fe (pure ≥ 96%; Carl Roth GmbH + Co KG, Karlsruhe, Germany) had a size < 149 μm. Z (Helitrophen, London, UK), in the form of clinoptilolite (90–92%), was a 100% natural volcanic mineral powder from Norway; ultrafine micronised with particle size < 20 μm.

### 2.2. Preparation of fPBA

Two grams of fish adhesive grains were mixed in 10 mL distilled water at room temperature for 30 min followed by additional stirring at 50 °C in a water bath for another 30 min. Then, 10% (based on the dry mass of the fish adhesive) of Cu, Fe, or Z was added to the fish adhesive solution, and heated for another 3 h at 50 °C. For comparison, unmodified fPBA was prepared in the same way, but without the additives. Corresponding to the type of the additive, samples were coded as fPBA, fPBA-Cu, fPBA-Fe, and fPBA-Z.

**Figure 1:**
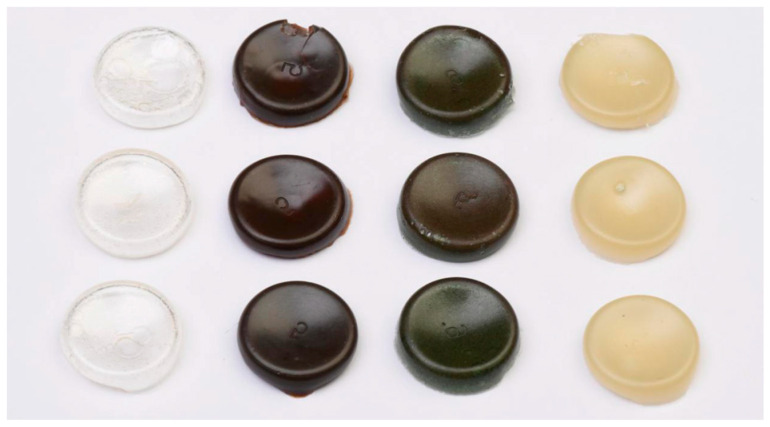
Samples of unmodified and modified fish fPBA, from left to right: fPBA, fPBA-Fe, fPBA-Cu, and fPBA-Z.

Mixtures were immediately poured into moulds. Triplicates were performed for each additive type. Samples were allowed to dry for 2 days in the conditioning room at 23 ± 2 °C and 50 ± 5% relative humidity (in accordance with ISO 291:2008 [40]) and analysed with different methods. Figure 1 shows examples of prepared samples of fPBAs for gravimetric measurements of humidity uptake.

### 2.3. Methods

#### 2.3.1. Sample Exposure in the Humidity Chamber

**Figure 2:**
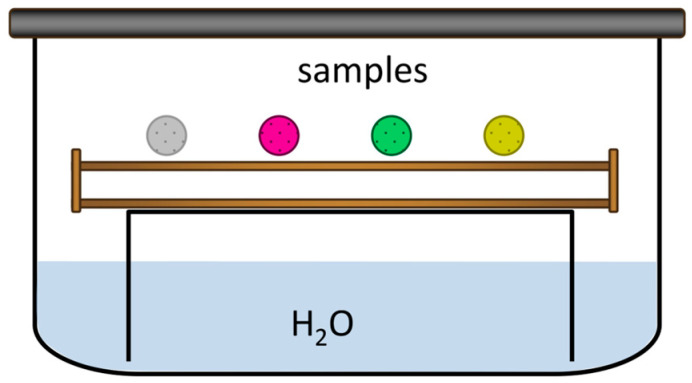
Well-sealed lid chamber for humidity uptake measurements.

The humidity chamber was prepared by pouring demineralised water into the bottom of a well-sealed container and kept in the conditioning room (ISO 291:2008 [40]). The room temperature was 23 ± 2 °C and the relative humidity measured in the container was 93 ± 2%. The samples were positioned just above the water surface (Figure 2) and exposed to this environment for 24 h.

#### 2.3.2. Dry Mass Content Determination

The dry mass content was determined by dividing the mass of unmodified and modified fPBA obtained after drying in the conditioning room (dry samples of fPBAs) by the mass of the wet sample (wet samples of fPBAs) multiplied by 100.

#### 2.3.3. Gravimetric Measurements of Humidity Uptake

The humidity uptake of the samples was determined by weighing the samples before and immediately after one day of exposure in the humidity chamber using a precision scale (XPR225, Mettler-Toledo GmbH, Greifensee, Switzerland, d = 0.00001 g) and the Equation (1):

(1) $$\mathrm{Moisture}\;\mathrm{uptake}\;(\%)=\frac{\left(m_{wet}-m_{dry}\right)}{m_{dry}}\cdot 100$$

where $m_{wet}$ means the mass of sample immediately after one day of exposure in the humidity chamber, and $m_{dry}$ is the mass of sample obtained after drying in the conditioning room.

#### 2.3.4. Thermogravimetric Measurements: TGA and DSC

Thermogravimetric analyses (TGA) were performed on the Discovery TGA (TGA 5500, TA waters instruments, New Castle, DE, USA). An average of 5.8 mg of sample was placed on aluminium pans and heated up to 800 °C at a heating rate of 10 °C/min in a nitrogen atmosphere. Sample and balance purge flow was set to 25 mL/min and 10 mL/min respectively. For each group (fPBA, fPBA-Cu, fPBA-Fe, and fPBA-Z), at least three measurements were conducted; the average values obtained from degradation curves are reported in the results. The degradation phase for onset and endset temperatures during the intensive degradation phase was selected from 175 °C to 600 °C. From the thermogravimetric curves, the following points were selected: (a) change in mass at 175 °C (m_175°C_), (b) degradation onset temperature (*T*_1_), start of the highest degradation phase, (c) maximum degradation temperature (*T*_2_), (d) degradation endset temperature (*T*_3_), end of intensive degradation phase, (e) mass loss at 600 °C, end of the test (m_600°C_), and (f) residual mass at the end of the test (m_residue_). The onset and endset temperatures were identified as the intersection of the initial and final tangential line along the curve of the slope, and the values referring to change in mass were extracted from the nearby end points. *T*_3_ represents the highest degradation temperature obtained from the degradation derivative slope.

Differential scanning calorimetry (DSC) analysis was performed on the Discovery DSC 25 (TA waters instruments, New Castle, DE, USA). Average samples of solidified film weighing 1.5 mg were placed in hermetically sealed aluminium pans. Analyses were conducted under nitrogen gas using the heat-cool method from room temperature to 400 °C, at a heating and cooling rate of 10 °C/min.

#### 2.3.5. Fourier-Transform Infrared Spectroscopic (FT-IR)

To determine differences in the prepared modified fPBA, the analysis was performed using an FTIR Spectrum Two spectrometer (PerkinElmer, Llantrisant, UK). Spectra were recorded in the range from 500 cm^−1^ to 4000 cm^ −1^, with a spectral resolution of 4 cm^−1^ and 16 scans. Spectra were obtained using ATR mode.

#### 2.3.6. Surface Tension Measurements: Contact Angle

Surface tension measurements using contact angle (CA) measurements were performed with an FTA 1000 DropShape Instrument B FrameSystem (First Ten Angstroms, Newark, NJ, USA). The instrument measures the contact angle between a solid surface and a liquid which is applied to the surface automatically using the incorporated dispenser. A drop of deionised water is placed on the sample and then the image is recorded. Three different measurements were performed in three different areas, with the average contact angle values reported as the result. All measurements were made at a room temperature of 23 ± 2 °C and relative humidity of 50 ± 5%.

#### 2.3.7. Surface Morphology

The distribution, the shape, and size of the powders inside fPBA were observed using a scanning electron microscope (SEM) JSM-IT500LV, Oxford Inca; Jeol, Peabody, Oxford Instruments Analytical, Pleasanton, CA, USA. The images were recorded in low vacuum mode at 12 Pa, accelerated voltage of 15 kV, and at a working distance of 10 mm. Images were obtained with retractable secondary electrons detector (BED-C).

#### 2.3.8. Visual Detection of Mould Growth

Adhesives were placed in closed containers at room temperature and observed for mould growth. When the first growth was visually observed, the image was recorded with a high resolution, full-frame DSLR, Nikon D850 digital camera (45.7 Megapixel BSI CMOS sensor with no optical low-pass filter, and an Expeed 5 image processor).

#### 2.3.9. Water Vapour Transmission

Water vapour transmission properties (vapour permeability tests) were performed based on the SIST EN ISO 7783:2018 [41]. To determine the water vapour permeability of the samples, unmodified and modified fPBAs were applied with a 100 micron spiral applicator (Erichsen, Hemer, Germany) onto a polyethylene tile, which is a standardised substrate with a defined pore size of 40 microns. The conditioning of the prepared samples before testing, as well as the subsequent testing of the samples themselves, took place in the conditioning room, at a temperature of 23 ± 2 °C and relative humidity of 50 ± 5%. The water vapour permeability of fPBA samples was determined by placing a saturated solution of NH4H2PO4 (ammonium dihydrogen phosphate, relative humidity inside the container 93%) in a container and closing it with a polyethylene tile applied with either unmodified or modified fPBA. The edges were waxed so that only moisture passed through the sample.

The water vapour resistance factor *μ* is a dimensionless value that indicates how many times greater the water vapour resistance of a material is compared with a layer of static air of the same thickness, temperature, and pressure. The *S_d_* value is the product of the water vapour permeability coefficient with the thickness (d) of the material in meters (*S_d_* = *μ* · *d*). A higher value means a higher vapour barrier of the material. From the obtained measurement, the water vapour diffusion equivalent *S_d_* was calculated. Water vapour permeability testing was performed for each sample in triplicate.

#### 2.3.10. Lapshear Strength of Modified fPBA Adhesive

Variations of modified fPBA (fPBA-Cu, fPBA-Fe, and fPBA-Z) along with reference fPBA were prepared as described under the preparation section and used to validate bondline strength following the EN 205:2016 [42]. Clean knot-free beech boards (*Fagusi sylvatica* L.) were planed prior to bonding. An average of 200 g/m^2^ adhesive spread rate was applied on the surface, and lamellas were pressed for 12 h under 1.2 MPa pressure using cold press (LZT-UK-30-L, Langzauner, Lambrechten, Austria). After pressing, glued lamellas were conditioned in a climate chamber (20 °C and 65% RH) prior to testing for at least 7 days. Lap shear tests were performed on a universal testing machine (Zwick Roell, Ulm, Germany) with a 100 KN load cell and a testing speed of 1 mm/min. For each type of adhesive modification, at least 10 replicants were tested. The testing area was measured with a calliper, and the maximum obtained force obtained during the testing was used to calculate the strength (*τ*).

## 3. Results and Discussion

### 3.1. Dry Mass Content Determination

The prepared samples as described in Section 2.3.2 were used for calculating the dry mass content of the various samples of fPBA (Table 1). Results show that dry matter content increased in order from fPBA to fPBA-Cu to fPBA-Z, and finally fPBA-Fe.

### 3.2. Gravimetric Measurements of Humidity Uptake

**Figure 3:**
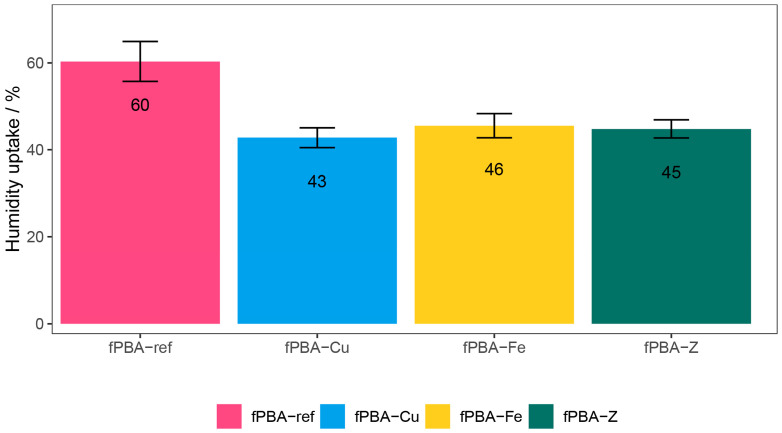
Moisture uptake of various fPBAs after 24 h exposure to 90 ± 5% relative humidity.

The moisture uptake of the unmodified and modified fPBA samples are presented in Figure 3.

The obtained results show that all three additives, Cu, Fe, and Z, successfully reduced the moisture uptake in the prepared modified fPBA samples. fPBA-Fe and fPBA-Z exhibited a 15% reduction in moisture uptake compared to the unmodified fPBA, and a 17% reduction was observed for fPBA-Cu. This observed reduction in moisture uptake could be explained as follows. Water insoluble raw powder materials most probably form an inorganic barrier within the organic film (fPBA), which makes it difficult for water vapour to penetrate. Outside media that can penetrate through the barrier films must traverse a more complicated route to reach the substrate [43,44]. It should be noted that moisture uptake (as well as other properties, i.e., stability, mechanical properties, and solubility) is also affected by the crystallinity of the proteins [45]. Higher crystallinity typically results in lower moisture uptake due to reduced free volume and fewer polar sites being accessible (lower solubility) for water interaction [46,47]. Since the origin of the investigated fish protein-based adhesive is precisely specified, as well as the previous steps during the processing of collagen, the crystallinity of the proteins of the tested samples was not determined.

### 3.3. Thermogravimetric Measurements—TGA and DSC

**Figure 4:**
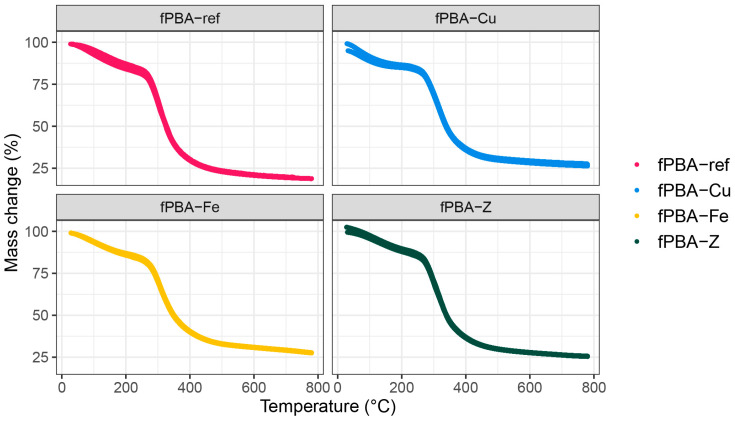
TGA curves of various fPBAs.

Table 2 shows the thermal decomposition of the unmodified and modified fPBAs, obtained from the TGA data presented in Figure 4.

The first degradation phase can be seen from the beginning of the test to 175 °C (m_175°C_), with average mass loss ranging from 11% to 14%. Perkasa et al. [48] reported that the degradation of fish collagen during this phase can be associated with the material’s formation history and its ageing process prior to the test. The degradation phase of the fish gelatine is attributed to the degradation and decomposition processes in lower molecular weight peptides, polysaccharides, and various forms of free and bound water [49,50]. Through hydrogen interactions, water can interact within and on the surface of the triple helix as well as around the protein chains [51]. At a temperature of 175 °C, the fPBA sample (unmodified) exhibited the largest mass loss (on average 14%) compared to the other samples, while the fPBA-Z had the least change in mass (on average 11%). The latter was likely favoured by the porous structure of the zeolite and the affinity for additional bound water, which could slow down the degradation in the multiple dehydration steps of the zeolite [52]. In the following second degradation phase, the onset temperature (*T*_1_) of the intensive degradation point was depicted. The fPBA sample exhibited the lowest onset temperature (*T*_1_), whereas fPBA-Fe had the highest, which was 2.5 °C higher than fPBA. The fPBA-Z and fPBA-Cu samples also showed a slightly higher onset point compared to unmodified fPBA. A change in the onset temperature of decomposition due to additives has been reported by other studies as well. For example, when copper slag was added to polylactide composite, there was a greater decrease in the onset temperature with increasing amounts of inorganic filler in the matrix, resulting in the reduced thermal stability of the material [49]. The temperatures of the most intense degradation (*T*_2_, maximum peak temperature) were similar for all samples and ranged between 305.7 °C and 308.1 °C. An exception was observed for the fPBA-Cu sample, which had a relatively high *T_2_* at 316.2 °C. The thermal events at about 300 °C were likely associated with the gelatine degradation process accompanied with the loss of amino acids [53]. The endset point (*T*_3_) represented the end of the intensive degradation phase and was about 363.9 °C for the unmodified sample. The endset temperatures for the fPBA-Cu, fPBA-Z, and fPBA-Fe samples were 4.3, 5.7, and 10.3 °C higher than the unmodified sample, respectively. Mass loss at 600 °C was the highest for the unmodified fPBA (76.8%), followed by the fPBA-Z (71.8%), fPBA-Cu (70.7%), and fPBA-Fe samples (68.7%). The results follow a similar trend to those determined by dry matter content in Table 1. However, results determined by TGA showed a slightly higher mass residue in the case of the sample with the addition of zeolites, presumably because all the absorbed water in the porous zeolite was also eliminated at higher temperatures. By the end of the test, up to 790 °C, the unmodified sample lost an additional 4.8%, with a final residual mass of 18.4%. However, for the modified samples, residual mass remained at 25.7, 27.2, and 27.6% for the fPBA-Z, fPBA-Cu, and fPBA-Fe samples, respectively. In previous studies, the residual mass when heating fish collagen to 600 °C [53] was reported at 26.6%, and 19.4% when heating to 800 °C [54]. The most visible effect of using fillers was the change in mass after reaching *T*_1_, and the end residuals. This property can be attributed to the addition of inorganic compounds in the mixture of modified samples, resulting in lower mass loss of the modified protein adhesive.

**Figure 5:**
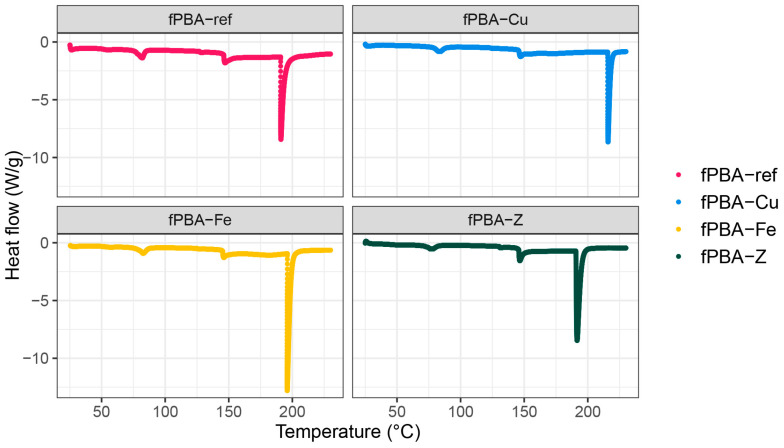
DSC curves of various fPBAs.

Table 3 shows the three values of fPBA degradation: (a) glass transition temperature (*T_g_*), (b) melting temperature (*T_m_*), and (c) denaturation temperature (*T_d_*), based on the results obtained from the DSC curves. The degradation curves can be also viewed in Figure 5.

The initial phase, in the temperature range of 79.0 °C to 82.7 °C, represents the glass transition temperature [55]. Samples fPBA-Fe and fPBA-Cu showed a higher glass transition temperature compared to the unmodified fPBA, while the peak temperature for sample fPBA-Z was the lowest among them all. Small molecules of zeolite mixed with fPBA lower the glass transition temperature. This observation is well-known and easily explained in free volume and entropy models of glass transition [56]. Additionally, the increased mobility of water molecules bound in the porous zeolite structure may cause the decrease in *T_g_*. The second phase, related to the melting point (*T_m_*), is also dependent on the thermal history and material ageing at room temperature [48]. The melting point temperatures ranged between 145.3 °C and 149.9 °C. The highest *T_m_* was obtained for the fPBA-Fe and fPBA-Cu samples compared to fPBA and fPBA-Z samples. The last phase, corresponding to the denaturation temperature (*T_d_*), is associated with the irreversible degradation (i.e., denaturation point) of collagen [48]. On average, *T_d_* typically ranged between 195.4 °C and 207.0 °C. Samples fPBA-Z and fPBA started denaturing at 206.7 °C and 207.0 °C, respectively, which was lower compared to fPBA-Fe or fPBA-Cu. According to the literature, fish binder in its natural state begins to denature at temperatures between 187 °C and 197 °C [48,55]. In our research, the unmodified fPBA sample reached a denaturation temperature of 196.5 °C. The slightly (~10 °C change in *T_d_*) higher degradation rate of the reference material compared to materials with added metal particles (Cu and Fe) can be attributed mainly to the reduced protein fraction in the sample. Due to the 10% decrease in protein content in the sample, the need for energy required for denaturation (*T_d_*) also decreases.

### 3.4. Fourier-Transform Infrared Spectroscopic Analysis of the Prepared Samples (FTIR)

**Figure 6:**
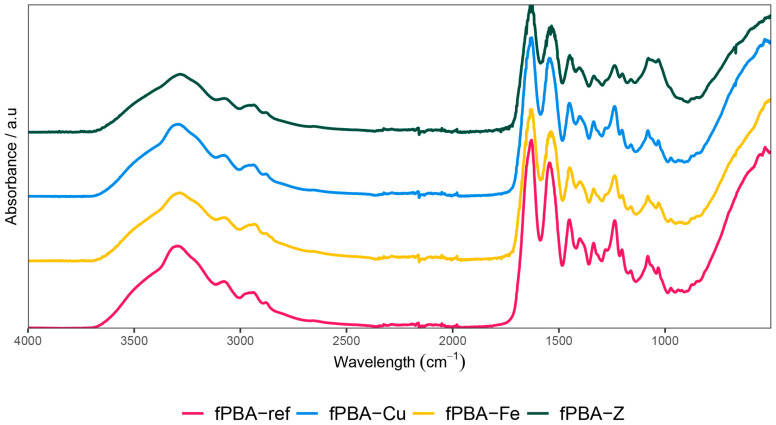
FTIR spectra of modified and unmodified fPBA.

Figure 6 shows the FTIR spectra of unmodified and modified fPBA. By comparing the spectra, one can see neither cleavage nor formation of new bonds, because the powder materials used are only embedded in the protein network of fPBA. The powder additives, thus incorporated in fPBA, are immobilised in fPBA during the drying/hardening phase and perform the function of barrier fillers there.

### 3.5. Contact Angle Measurements (CA)

**Figure 7:**
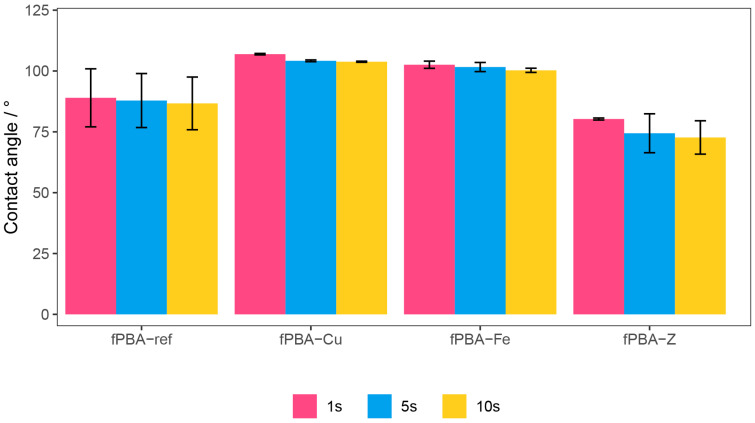
Contact angle values of samples obtained after 1 s, 5 s, and 10 s.

Hydrophilicity or hydrophobicity is the degree to which a surface exhibits affinity or lack of affinity for water. Figure 7 shows the CA measurement results for the dry unmodified and modified fPBA samples.

The CA measurements after 1 s, 5 s, and 10 s for fPBA-Fe and fPBA-Cu exhibited higher hydrophobicity than CA values for the unmodified fPBA and the modified sample fPBA-Z. Depending on the water contact angle, we can distinguish between hydrophilic, CA < 90° (unmodified fPBA and the modified sample fPBA-Z), and hydrophobic, CA > 90° (fPBA-Fe and fPBA-Cu), surfaces [57,58].

The contact angle decreased most with time for the fPBA-Z sample, which may be the result of the absorption of dripping water into the porous structure of the zeolite. The high porosity (clinoptilolite 34%) [59] of zeolites means they can retain water molecules up to 60% of their weight. Therefore, water in their pores can steadily evaporate or be reabsorbed without damaging the crystalline structures. It is a surface reaction, which is why, according to the CA measurements, zeolite has an even lower hydrophobicity than the unmodified fPBA. This was not confirmed with gravimetric measurements when testing exposure in high humidity. However, it can be concluded that zeolites did not increase the hydrophobicity of the sample.

### 3.6. Surface Morphology (SEM)

**Figure 8:**
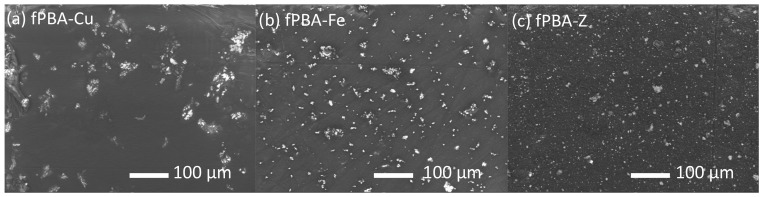
SEM images of the modified samples. (**a**) fPBA-Cu, (**b**) fPBA-Fe, and (**c**) fPBA-Z.

**Figure 9:**
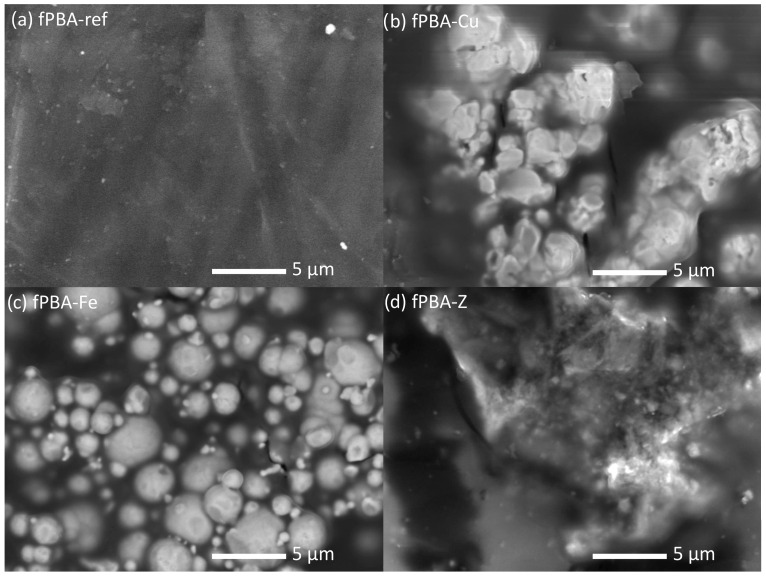
SEM images of unmodified (**a**) and fPBA modified with (**b**) Cu, (**c**) Fe, and (**d**) zeolite additive.

Figure 8 shows the distribution of Fe, Cu, and Z solid particles in fPBA. It is evident that the Fe and Z particles are evenly distributed over the fPBA matrix, whereas with the addition of Cu, the particles appear in larger aggregates/agglomerates. The detailed shape, morphology, and size of the powder materials inside the fPBA are shown at a higher magnification in Figure 9. The SEM image of unmodified fPBA shows that the surface is solid and smooth (Figure 9a). The change in surface morphology could also affect the hydrophobicity of the sample [60], which depends on the type and size of the particles and their tendency to agglomerate in the given system, along with the amount of powder additive. The more difficult dispersion of smaller particles in the liquid phase could lead to a greater tendency for agglomeration. This is particularly evident when comparing the samples with Cu (Figure 9b) and Fe (Figure 9c) added. In terms of size, the Cu particles are smaller than the Fe particles (see Section 2.1.). However, the results show that the Cu particles appear to be larger than the Fe particles. In our system, Cu particles seem to have had a higher tendency to agglomerate than Fe particles, which were more spherical in shape and better dispersed in the matrix, and consequently showed a lower tendency to agglomerate.

Figure 9c shows that the fPBA-Fe sample has the most uniform shape of the distributed Fe particles; they are spherical and of varying sizes, up to a maximum of 5 µm. In contrast, Cu particles within the fPBA-Cu sample are more irregularly shaped and compacted (Figure 9b). A clear surface morphology was not achieved with the addition of zeolite (Figure 9d).

However, the homogeneity of the distribution of powders across the matrix indicates slightly better tensile shear strength of the sample, and Fe particles were included more evenly. Due to their larger specific surface area, smaller particles also consume more liquid medium to wet their surface, which can be reflected in a lower remaining amount of the liquid part of the protein and thus slightly better water vapour transmission properties.

### 3.7. Visual Detection of Mould Growth (Non-Quantified, Indicator Test)

**Figure 10:**
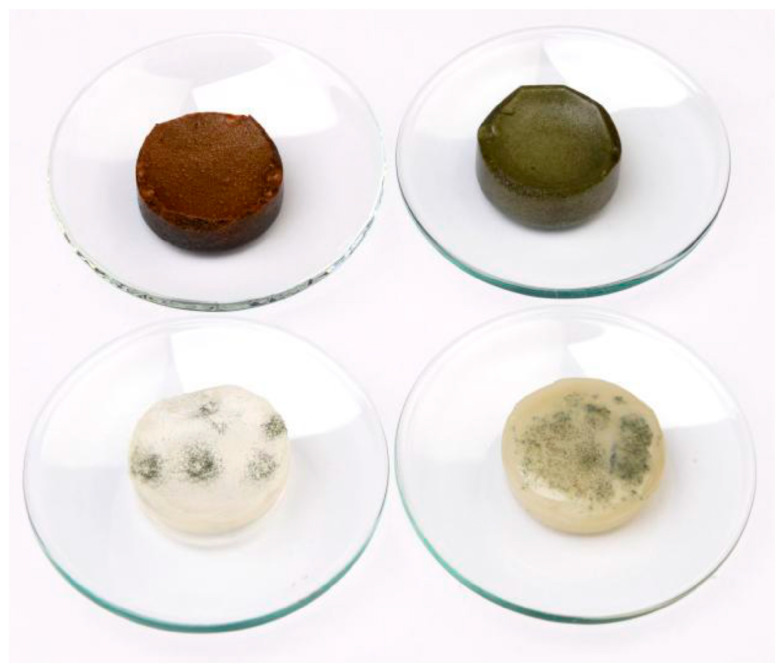
Visual detection of mould growth after 14 days at room temperature.

Figure 10 shows an image of mould growth after 14 days at room temperature. On the fPBA and fPBA-Z samples, mould appeared after 14 days, while no mould was detected on the fPBA-Cu or fPBA-Fe samples during this time.

These results are predictable, as Cu is known to have an antimicrobial effect. This also applies to Fe, where iron(III) oxide and hydrogen can be formed, which can exhibit antimicrobial properties. On the other hand, zeolite is hydrophilic with a crystal network with open voids in the form of cages and tubules. Those voids can adsorb various substances and a lot of water [61], which may provide suitable growth conditions for mould [62].

### 3.8. Water Vapour Transmission

Table 4 shows weight loss due to water permeability through fPBA and modified fPBA films after 3 h, 6 h, 24 h, and 48 h. Although there are no significant differences between the results, we can see minimal deviations in the modified samples from the reference sample. Results show that unmodified fPBA is quite permeable to water vapour. Nevertheless, we can give a possible interpretation of the results of the liquid water permeability test for the 10% addition of Cu, Fe, and Z powder, which slightly changes the liquid water vapour permeability through the network/film and thus shows a certain trend. Measurements show the difference between the vapour permeability of fPBA-Fe and fPBA-Cu compared to the unmodified fPBA and fPBA-Z. Particles of Fe and Cu can partly fill the pores of the base and can act as a barrier to the passage of water vapour. In the case of fPBA-Z, water can uniformly evaporate or be reabsorbed from the pores of the zeolite without damaging the crystal structure [63,64], which maintains the vapour permeability. It has been proven that adsorption on zeolites is characterised by Langmuir-type isotherms, which describe the maximum amount of water vapour that can be retained at a given pressure and temperature [65]; therefore, the Langmuir adsorption isotherm is used to describe the balance between the adsorbate and the adsorption system, where the adsorption of the adsorbate is limited to one molecular layer [66,67]. When such a sample is taken from a chamber with an approx. relative humidity of 93% and is transferred to an environment with a relative humidity of 50%, this “one-layer” of absorbed water quickly passes from the pores of the zeolite and diffuses back into the environment due to the already described properties of zeolite [66]. Therefore, fPBA-Z showed a decrease in water absorption in the water uptake test.

### 3.9. Lapshear Strength of Modified fPBA Adhesive

**Figure 11:**
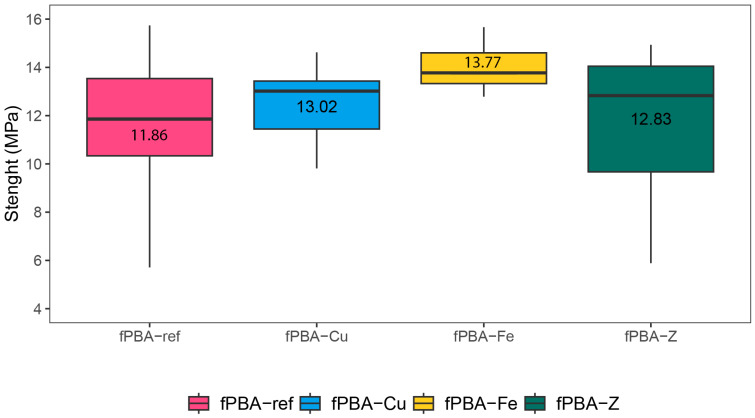
Tensile shear strength of modified and unmodified fPBAs.

Mean strength values of tensile shear strength (τ) for the tested specimens were 12.1, 13.8, and 11.4 MPa for fPBA-Cu, fPBA-Fe, and fPBA-Z, respectively (Figure 11). The mean strength for the reference adhesive (fPBA) was 11.0 MPa. This indicates that modification of the adhesive did not have negative impacts on the bonding properties in this setup, which was the main goal of those tests. Out of the modified adhesives, the highest τ values were obtained for the fPBA-Fe sample. In the cases of fPBA-Fe and fPBA-Cu modification, lower moisture uptake was recorded compared to fPBA and fPBA-Z, and τ values from mechanical tests were found to be higher for these two types. The contribution of Fe and Cu filler may beneficially work in (a) interlocking between the particles and wooden surface, (b) lowering moisture content in the adhesive bondline, and (c) improving the mobility of adhesive for better application on the surface. The use of fillers in adhesives is often practiced for improving various properties of adhesives, such as cost, mechanical properties, viscosity etc. Often, when added in sufficient proportions, adhesive characteristics can be tailored in favour of desired needs [68]. Similarly, the literature reports improvements in bondline strength with epoxy enhanced Cu nanoparticles resin-glued alu alloys [69,70] or the addition of iron oxide to hot-melt adhesives, which also resulted in slightly improved strength values [71]. The differences in τ values, in the case of fPBA, may also be attributed to material variability or the manufacturing process; therefore, more research parameters should be studied to understand the origin of potential differences.

As mentioned, in this study, higher values of τ were obtained for the fPBA-Fe modification, and smaller differences were observed in τ for fPBA-Cu and fPBA-Z. S.W. Werneke et al. found that transition metals play an essential role in the performance of the adhesive, as the removal of iron and related transition metals from the adhesive increased the solubility of the adhesive. Electrostatic interactions and other coordinate-covalent bonds play a role in metal-catalysed oxidations, as they may explain the ability of adhesive proteins to bind to the metal and may participate in the initial interaction during crosslink formation [47].

## 4. Conclusions

This study addressed polymer stability by limiting hygroscopicity, which is the main weakness of natural fish protein adhesives. Polymer stabilization was studied by enhancing hydrophobicity with the addition of different sized solid iron (Fe), copper (Cu), and zeolite (Z), as well described in Section 2.1.

While the formation of new bonds is not visible from FTIR analysis, fPBA showed reduced hygroscopic properties and improved hydrophobicity due to the additives, especially with fPBA-Fe and fPBA-Cu. Contact angle measurements showed a hydrophobic surface in fPBA-Fe and fPBA-Cu and a hydrophilic surface in the unmodified fPBA and fPBA-Z samples. Due to the hydrophilicity of zeolite and its ability to absorb and retain water in the microporous structure, this sample had the most mould growth. The appearance of mould was also observed on the unmodified fPBA, while no mould was detected on the fPBA-Fe and fPBA-Cu samples. The zeolite network, with its open voids, allows water absorption, water vapour passage, evaporation, and reabsorption. Both the fPBA and fPBA-Z samples exhibited slightly higher water vapour transfer capacity compared to fPBA-Fe and fPBA-Cu. fPBA-Fe and fPBA-Cu contain hydrophobic Fe and Cu particles, which act as barrier fillers in the fPBA protein network. They fill spaces and impede moisture diffusion, significantly delaying the penetration time of water vapour. Additionally, the uneven distribution or agglomeration of barrier fillers within the formed protein film can result in large areas without barrier particles, impacting water vapour permeability. SEM analysis showed a good distribution of Fe, Cu, and Z particles in the protein matrix. The bondline strength of the modified adhesive was determined by the tensile shear strength of the lap joints, which showed that the modifications to the adhesive did not negatively affect the bond properties in this set-up, which was the main goal of those tests.

To optimise the use of protein-based adhesives, understanding their temperature-dependent behaviour is crucial. TGA and DSC analyses were conducted on all samples, revealing temperature effects even below 100 °C. Thermal effects up to around 200 °C were observed on PBAs, involving dehydration, water binding, and the degradation of peptides and polysaccharides. Unmodified fPBA showed the highest mass loss at 175 °C, while fPBA-Z exhibited the lowest mass change, indicating the highest thermal stability. The fillers notably influenced mass change after 175 °C, providing essential data for using protein adhesive.

## Figures and Tables

**Table 1:** Compositions of the fPBAs.

Samples	Wet Sample Mass (g)	Dry Sample Mass (g)	Dry Content (%)
fPBA	0.385 ± 0.07	0.075 ± 0.01	19.398 ± 0.06
fPBA-Cu	0.415 ± 0.07	0.089 ± 0.01	21.316 ± 0.07
fPBA-Fe	0.374 ± 0.05	0.081 ± 0.01	21.730 ± 0.01
fPBA-Z	0.367 ± 0.02	0.078 ± 0.00	21.377 ± 0.04

**Table 2:** Mass change at 175 °C (m_175°C_), onset (*T*_1_), peak degradation (*T*_2_), and endset temperature, and residual mass at 600 °C (m_600°C_) and at the end of the test (m_residue_).

Samples	m_175°C_ (%)	*T*_1_ (°C)	*T*_2_ (°C)	*T*_3_ (°C)	m_600°C_ (%)	m_residue_ (%)
fPBA	14.0	272.4	305.7	363.9	76.8	18.4
fPBA-Z	11.1	274.4	308.1	369.6	71.8	25.7
fPBA-Cu	13.0	273.7	316.2	368.2	70.7	27.2
fPBA-Fe	12.8	274.9	306.7	374.2	68.7	27.6

**Table 3:** DSC results of glass transition temperature (*T_g_*), melting temperature (*T_m_*), and denaturation temperature (*T_d_*).

Samples	*T_g_* (°C)	*T_m_* (°C)	*T_d_* (°C)
fPBA	82.7	145.3	196.5
fPBA-Fe	83.7	149.9	206.7
fPBA-Cu	84.9	147.3	207.0
fPBA-Z	79.0	145.6	195.4

**Table 4:** Water vapour transmission properties of fPBA, fPBA-Cu, fPBA-Fe, and fPBA-Z.

	s_d__3 h [m]	s_d__6 h [m]	s_d__24 h [m]	s_d__48 h [m]	${\bar{s}}_{d}$ [m]
fPBA	0.058	0.056	0.056	0.056	0.056
fPBA-Cu	0.056	0.050	0.054	0.049	0.052
fPBA-Fe	0.053	0.051	0.056	0.054	0.054
fPBA-Z	0.057	0.058	0.063	0.057	0.059

## Data Availability

The original contributions presented in the study are included in the article, further inquiries can be directed to the corresponding author.
